# The frantic play of the concealed HIV envelope cytoplasmic tail

**DOI:** 10.1186/1742-4690-10-54

**Published:** 2013-05-24

**Authors:** Eveline Santos da Silva, Martin Mulinge, Danielle Perez Bercoff

**Affiliations:** 1 Laboratory of Retrovirology, CRP-Santé, 184 Val Fleuri, Luxembourg, L-1526, USA

**Keywords:** HIV-1, Lentiviruses, Envelope gp41 cytoplasmic tail, Envelope trafficking, Viral assembly, Envelope intracellular interacting factors

## Abstract

Lentiviruses have unusually long envelope (Env) cytoplasmic tails, longer than those of other retroviruses. Whereas the Env ectodomain has received much attention, the gp41 cytoplasmic tail (gp41-CT) is one of the least studied parts of the virus. It displays relatively high conservation compared to the rest of Env. It has been long established that the gp41-CT interacts with the Gag precursor protein to ensure Env incorporation into the virion. The gp41-CT contains distinct motifs and domains that mediate both intensive Env intracellular trafficking and interactions with numerous cellular and viral proteins, optimizing viral infectivity. Although they are not fully understood, a multiplicity of interactions between the gp41-CT and cellular factors have been described over the last decade; these interactions illustrate how Env expression and incorporation into virions is a finely tuned process that has evolved to best exploit the host system with minimized genetic information. This review addresses the structure and topology of the gp41-CT of lentiviruses (mainly HIV and SIV), their domains and believed functions. It also considers the cellular and viral proteins that have been described to interact with the gp41-CT, with a particular focus on subtype-related polymorphisms.

## Review

### Background: Env and the entry process

The main target cells for the Human Immunodeficiency Virus type 1 (HIV) and Simian Immunodeficiency Virus (SIV) are CD4-expressing cells, namely CD4+ T lymphocytes, dendritic cells (DCs) and macrophages. The first step of the viral cycle, involving anchoring to the target cell membrane and entry, is mediated by the envelope glycoprotein (Env). Env is composed of two non-covalently linked subunits, SU (surface glycoprotein or gp120) and TM (transmembrane glycoprotein or gp41), displayed as heterotrimeric spikes on the surface of virions and infected cells [1-6]. The two Env subunits are the products of the proteolytic cleavage of a highly glycosylated gp160 precursor protein by the cellular protease Furin in the Golgi apparatus. gp120 is responsible for binding to the CD4 receptor and the coreceptor expressed at the cell surface of target cells. gp120 is composed of several variable and constant regions; it comprises a heavily glycosylated outer domain and an inner domain, which are linked by a structure called the bridging sheet. Mature Env thus forms a cage-like structure with an interior void; this cage-like structure is believed to restrict antibody access [7]. gp41 is non-covalently linked to gp120 and the N-terminal ectodomain of gp41 contains a hydrophobic, glycine-rich fusion peptide that mediates fusion of the viral and target cell membranes. Binding of gp120 to the CD4 receptor induces conformational changes that expose the third hypervariable region (V3 loop) of gp120, which in turn binds one of the co-receptors, CCR5 or CXCR4 [8-12]. Binding to the co-receptor triggers further conformational changes within gp41, which adopts the so-called “fusion-active” state required for bringing the viral and cellular membranes into close contact and allowing fusion. These conformational changes involve two leucine zipper-like motifs, the heptad repeat 1 (HR1) and heptad repeat 2 (HR2): HR2 folds in an anti-parallel fashion onto the pre-formed trimer of HR1 helices, allowing HR1 and HR2 to assemble into a highly stable antiparallel six-helix bundle structure, which juxtaposes the viral and cellular membranes, and allows fusion of the viral and cellular membranes [2,3,5,6,13].

Most of the Env precursor is retained in the endoplasmic reticulum (ER) or the cis-Golgi compartments and only a small proportion progresses through the trans-Golgi network (TGN) and reaches the cell membrane [14-17]. As it is synthesized and oligomerizes in the rough ER (RER), Env is extensively N-and O-glycosylated, sulfated and plamitoylated concomitantly with its translation and transit through the TGN [1,18-24] reviewed in [25]. Glycosylation of gp120 accounts for nearly half of its molecular mass. This substantial glycosylation is believed to ensure correct protein folding and to favor immune escape by providing a glycan shield against neutralizing antibodies. As the disease progresses, viral strains lose potential N-glycosylation sites (PNGs) and display notably reduced glycosylation; these strains are consequently less well shielded from neutralizing antibodies [26,27]. Nonetheless, such viral strains appear late during infection, concomitantly with the decline in CD4+ T cell counts, such that the immune system is unable to trigger an efficient B-cell response; consequently, evading the immune system is no longer necessary [28].

Env is the most variable region of the HIV genome, and inter-subtype diversity is typically up to 25-35%. This variability is not evenly distributed throughout the *env* gene: the gp120 ectodomain is the most variable, the gp41 ectodomain is the least variable, and the gp41 cytoplasmic tail (gp41-CT) lies somewhere in between, with an intermediate level of sequence diversity and phylogenetic evolution [29]. Experimental data obtained from peptides and by sequence analysis and modeling indicate that the structural and physicochemical properties of the gp41-CT are surprisingly highly conserved despite the sequence variations. Presumably, this reflects constraints on its evolution imposed by crucial functional roles. However, these roles have mostly been explored using the NL4-3 reference strain or synthetic peptides.

### Aims of the review

The ectodomain of Env has been the subject of much interest and substantial investigations. The structural and functional characterization of the HIV and SIV gp41-CTs has received far less attention, and indeed, there are numerous contradictory results. Early studies of the gp41-CT mainly addressed the immunogenic properties of the Kennedy epitope, or the viroporin properties of the α-helical lentiviral lytic peptides LLP-1, LLP-2 and LLP-3 and their role in viral incorporation and infectivity. The discovery that the gp41-CT interacts with the p55^Gag^ precursor during viral assembly and ensures Env incorporation into virions has led to a reconsideration of the functional importance of this domain. It was, nevertheless, only very recently that its role in modulating Env expression and viral infectivity through a plethora of interactions with cellular partners started to be appreciated. Three recent reviews are relevant: they address the role of the HIV-1 gp41-CT in Env trafficking, incorporation and viral assembly [25], the biophysical properties and topology of the gp41-CT [30] and the interactions of the gp41-CT with cellular factors [31]. Parallel spectacular advances in cell and virion imagery, and very recent studies on the biophysical characteristics of mature and immature virions based on Atomic Force Microscopy (AFM) [32] and STED microscopy [33] provide new insights into the contribution of the gp41-CT to viral fusion efficiency. Here, we review current knowledge on the gp41-CT and its believed structure and function as a regulator of viral infectivity. In particular, we focus on variability and subtype-related specificities, as most studies on the gp41-CT are based on the NL4-3 reference and mutants thereof, in sharp contrast to studies on the Env ectodomain.

### Structural determinants and topology of the gp41-CT

#### Structure of the gp41-CT

**Figure 1:**
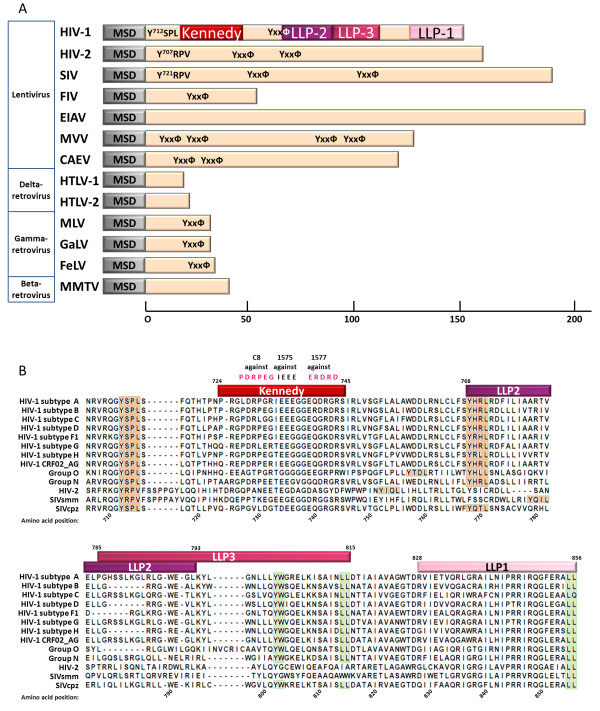
**Lengths of the cytoplasmic tails of various retroviruses and sequence alignment of different HIV-1 subtypes, clades and types.** (**A**) Comparison of the lengths of the gp41-CT of Human Immunodeficiency Virus type 1 (HIV-1), HIV type 2 (HIV-2), Simian Immunodeficiency Virus (SIV), Feline Immunodeficiency Virus (FIV), Equine Infectious Anemia Virus (EIAV), Maedi-Visna Virus (MVV), Caprine Arthritis Encephalitis Virus (CAEV), Human T-lymphotropic Virus type I (HTLV-1), Human T-lymphotropic V*type* 2 (HTLV-2), Mouse Mammary Tumor Virus (MMTV), Murine Leukemia Virus (MLV) and Gibbon Ape Leukemia Virus (GaLV); conserved YxxФ motifs (where Ф represents a hydrophobic residue) are highlighted. (**B**) Sequence alignment of the gp41-CT from various lenviviral (HIV and SIV) types and subtypes: HIV-1 subtypes A (92UG037), B (HXB2), C (ETH2220), D (SE365), F1 (93BR020), G (175), H (90CF056), CRF02_AG (93TH065), clade O (ANT70) and clade N (YBF106), HIV-2 (UC2), SIVcpz (Ptt_04CAM155) and SIVsmm (H635). AA positions are aligned against the NL-3 reference used in most studies of the gp41-CT. Lentiviral lytic peptide (LLP) domains, Kennedy epitopes and antibodies directed against them are shown. Conserved YxxФ motifs are highlighted in orange; conserved dileucine motifs, and motifs involved in cell trafficking are highlighted in green.

The gp41-CT of lentiviruses is unusually long (~150 amino acids) and substantially longer than the tails of other retroviruses (< 50 AA) [34] (Figure 1A). Although amino acid (AA) sequence identity is not conserved, both secondary structures and various endocytic and trafficking motifs are highly conserved across HIV-1 clades and even among lentiviruses in general [29,30] (Figure 1B). Immediately downstream from the membrane-spanning domain, gp41-CT has a loop containing the highly immunogenic Kennedy epitope (KE) which is not believed to adopt any particular conformation. The KE in fact harbors three consecutive epitopes (P_728_DRPEG_732_, I_733_EEE_736_ and E_739_RDRD_743_) [35]. Antibodies against the KE (SR1) display neutralizing activity against the infectivity of free virus and impair cell-to-cell spread by inhibiting Env-mediated fusion [36-38]. ERDRD is a conformational epitope and elicits production of neutralizing antibodies; the linear IEEE epitope seems to deflect the humoral response to favor viral immune escape from a neutralizing response against ERDRD [35].

**Figure 2:**
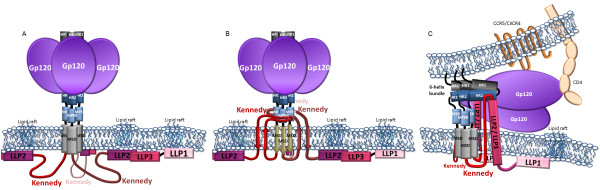
**Schematic representation of the topology of the gp41-CT. A**. Conventional model, in which gp41 spans the membrane once and the membrane-spanning domain (MSD) is an α-helix (boxed). **B**. Alternative model in which the gp41-CT crosses the membrane three times via antiparallel β-sheets (arrows) and the KE is exposed extracellularly. Adapted from Steckbeck et al. PlosOne 2010 [71]. **C**. LLP-2 is a membrane-traversing peptide or a “carrier” peptide and may interact with the HR1-HR2 6-helix bundle at the moment of fusion. In this conformation, the KE would also traverse the plasma membrane. Adapted from Lu et al. J Biol Chem 2008 [60].

The KE is followed by three α-helical motifs named lentiviral lytic peptides (LLP)-2 (AA 768 to 793 of NL4-3), LLP-3 (AA 785 to 807) and LLP-1 (AA 828 to 856) [39-41] (Figure 1B). LLP-3 partly overlaps LLP-2, and has been less studied than the other two LLP domains. Biochemical analyses with LLP peptides indicate that although primary amino acid identity is not conserved across clades (with the exception of the more conserved LLP-1), these domains adopt an α-helical structure in a membrane-mimetic environments and random coil conformations in an aqueous environment [29,30]. Early studies focused on determining the role and biochemical properties of these domains; they were based on synthetic peptides and three-dimensional computer modeling [39,40,42] or point mutations and truncation mutants of the gp41-CT, analyzing Env incorporation into virions [43-46] (detailed below), viral infectivity [47] and syncytia formation [46]. In the peptide form, the LLP domains interact with the plasma membrane [41,48,49] and thereby decrease bilayer stability [41,50] or alter the ionic permeability of membranes [48,50,51]. Intracellular localization studies suggested that the LLP helices may lie adjacent to and line the plasma membrane [49] (Figure 2A). These observations have led to the idea that the LLP domains are involved in fusogenicity, immunogenicity, and in conformational regulation of Env accompanying viral maturation and infectivity (see below, section ‘Viral stiffness, viral maturation and infectivity’).

#### Functional domains of the gp41-CT

Mutational analyses and work with truncation mutants of specific LLP-1 or LLP2 domains indicate varying Env expression and processing phenotypes, depending on the extent of the truncation and on the cell type. Despite subtle differences in the extent of the truncations studied, it appears that deleting the C-terminal domain of LLP-1 diminishes virion infectivity and cell-to-cell spread, due to decreased Env incorporation into virions [43-46,52] and to diminished Env expression and stability of the gp41/gp120 heterodimeric complex both at the cell membrane and in virions [44,53]. Env protein synthesis and processing in transfected HeLa or HEK293 cells is however unaffected by truncations of the C-terminal domain of LLP-1 [43,46,53]. Larger truncations, i.e. truncation of the full gp41-CT, or the region downstream from LLP-3 (i.e. deleting upstream from LLP-1), or even LLP-1 deletions or point mutations in this region dictate Env fusion capacity of mature [46] and immature virions [47,54]. Some authors attributed this phenotype to failed Env multimerization, a prerequisite for viral infectivity [55,56]; others proposed that truncation of LLP-1 induces modifications of the helical structure of LLP-2 and/or alterations in the structure of the Env ectodomain, suggesting an inside-out regulation of viral infectivity and fusion capacity [46,47,54,57,58]. Biochemical studies involving the use of synthetic peptides found that viral infectivity is dependent on interactions of LLP-1 and LLP-2 with the cell membrane [49,56,58] and/or with regions of the gp41 ectodomain, and particularly the HR1-HR2 6-helix bundle [29,59,60]. For SIVmac239, truncations of the gp41-CT affected the fusion capacity of Env in HeLa-CD4 cells by changing the conformation of the Env gp41 ectodomain [61] or the amount of gp120 on the surface of the virion due to an unstable gp120-gp41 association [62]. Indirect support for gp41-CT affecting the conformation of the gp41 ectodomain has been provided by studies showing that truncations of the gp41-CT affect the conformation of gp120 [57] and of the gp41 ectodomain [63]; also, such truncations interfere with the accessibility of these domains to antibodies targeting various epitopes including the CD4 or co-receptor binding sites [57] or the Membrane Proximal External Region (MPER) (neutralizing antibodies such as 2F5 and 4E10) [63]. In addition, a L49D mutation in the matrix protein (MA) of the p55^Gag^ precursor, which interacts with the gp41-CT (see below, section ‘Viral assembly, Env incorporation into virions and viral infectivity’), is associated with decreased amounts of gp120 at the surface of the virion without affecting gp41 incorporation [64]. This suggests that modifications to the MA/gp41-CT interaction may affect the level of gp120 incorporation [64]. This defect could be reversed by truncating the gp41-CT or disrupting the Y_712_SPL endocytic motif (i.e. impairing Env down-regulation, see section ‘Env trafficking and sorting through the TGN’). This implies that altering the interaction between p55^Gag^ and the gp41-CT affects the conformation of gp41 and thereby the incorporation of gp120 into virions [64]. Although these reports are descriptive and the proposed mechanisms are divergent, these studies agree that the gp41-CT may modulate the conformation of the Env ectodomain [46,47,54,57,58] and the stability of the 6-helix bundle [29,59,60] thereby affecting fusion capacity and sensitivity to neutralization [57,63].

The dileucine motifs that span the gp41-CT LLP helices play a role in viral replication, as has been demonstrated through a cumulative mutagenesis approach [65]. Bhakta et al. reported a cumulative effect on the infectivity of free virions and on viral propagation that did not correlate with modifications in viral incorporation or expression at the cell membrane [65]. Importantly, disruption of individual dileucine motifs had only a moderate impact, whereas the cumulative effect was substantial, suggesting a degree of flexibility, and tolerance of mutations of single dileucine motifs [65]. This study reconciles early and more recent reports on the role of the gp41-CT, as it suggests that impaired replicative capacity and viral spread may result from disrupting the hydrophobic properties of the LLP-2 α-helix: this disruption may impair interactions with the cell membrane and/or membrane translocation and interaction with the HR1-HR2 6-helix bundle [65]. Another possible explanation is that altering the biochemical amphipathic properties of the gp41-CT, or simply disrupting the LL dileucine domains, interferes with Env/Env interactions and coalescence [33,55], thereby affecting infectivity. This issue requires further investigation. Intriguingly, a highly conserved seven AA insertion (NSLKGLR) between R_787_ and R_788_ (LLP-2 domain) is found in many subtype C strains where the dileucine motifs spanning the gp41-CT are disrupted (ESS, MM and DPB, unpublished observation). The two supplementary α-helical turns result in three arginine residues being aligned side-by-side on the hydrophilic face of the α-helix. Whether adding these two α-helical turns to LLP-2 modifies its biophysical properties remains to be established.

The gp41-CT also harbors many functional determinants, including internalization motifs, for example: two YxxΦ motifs (Φ representing a hydrophobic AA, generally L in HIV and V in SIV), one immediately downstream from the transmembrane domain (membrane-proximal YxxΦ) and one within LLP-2; a Y_802_W_803_ diaromatic motif within LLP-3; two inhibitory sequences named IS1 (AA 750 to 763 of HXB2) and IS2 (AA 764 to 785); and the various dileucine (LL) motifs spanning the gp41-CT (Figure 1B). The role of these motifs in Env trafficking and protein-protein interactions is considered in more detail below (section ‘Interactions of the gp41-CT with cellular factors and intracellular regulators’).

#### Topology of the gp41-CT

gp41 is generally thought to be a type I membrane-spanning protein, with one α-helical membrane spanning domain (MSD) (AA 671–705) [66]. However, alternative models have been proposed, in which the gp41-CT lies partly outside the virus or the infected cell, crossing the membrane three times [38,60,67-71] (Figure 2B and C).

#### The Kennedy Epitope

Serum from HIV-infected individuals contains antibodies against epitopes within the KE. This has led to the hypothesis that this region is exposed extravirally and extracellularly, consistent with gp41 spanning the membrane three times rather than once [38,67-70]. In this model, the membrane-spanning domains form three antiparallel β-sheets [30,71] (Figure 2B). In support of this model, IgG against the gp41-CT-encoded epitopes P_728_DRPEG_732_, I_733_EEE_736_ and E_739_RDRD_743_ bound and neutralized free virions, and protease digestion inhibited antibody binding [69]. Other studies showed that neutralization by these antibodies extended to cell-to-cell fusion [38]. However, this model places the Y_712_SPL internalization motif outside the cell such that it would not allow and ensure an interaction of Env with cellular partners (see section ‘Env trafficking and sorting through the TGN’). Moreover, experiments performed with amphotericin B methyl ester (AME), a cholesterol-binding compound that interferes with virion-cell membrane fusion, showed that AME-resistant HIV variants contained mutations within the gp41-CT that create a cleavage site for the viral protease within the Y_712_SPL motif [72]. By cleaving the gp41-CT, HIV mimics the shorter gp41-CT of other retroviruses to restore Env fusogenicity [72,73]. (In the case of SIV, AME-resistant SIV gp41-CT displayed a premature STOP codon [72], and the topology of the gp41-CT does not affect its capacity to arise). Cleavage of the gp41-CT by the viral protease is not compatible with this epitope lying outside the virion. A more dynamic view of gp41-CT topology has recently been proposed to reconcile these observations [71] (reviewed in [30]). This model posits that the KE can adopt various distinct topologies: inside the virion, the KE would lie intravirally (Figure 2A), whereas in transfected/infected cells, the KE would lie outside the cell (Figure 2B) [71]. In support of this model, the authors present evidence that the gp41-CT of all HIV clades and of SIV share a highly conserved enrichment in arginine over lysine [29,40]. Arginine-rich peptides may be located within lipid environments and may readily cross biological membranes due to their guanidium group; they can also carry soluble proteins across membranes [74-77]. This dynamic model is compatible with the viral protease cleaving the gp41-CT in the virion under AME selective pressure. Note, however, that the authors compared the location of the KE in virions (i.e. in entities containing MA) with that in cells transfected with a construct encoding Env only rather than in cells transfected with a full HIV coding sequence. The interaction between the gp41-CT and p55^Gag^ (see section ‘Interaction of the gp41-CT with Gag’) is not taken into account in this model and it may not be excluded that the absence of Gag in the experimental setup may bias the topology of the gp41-CT, including that of the KE. 

The dynamic topology model still predicts that the Y_712_SPL internalization motif is outside the cell: this is not consistent with the regulation of Env expression at the plasma membrane of infected cells through adaptor protein (AP)-mediated internalization (see section ‘Env trafficking and sorting through the TGN’). The authors [71] do not address this point. This dynamic model has been opposed on the grounds that the model for a dynamic intra/extra viral localization of the KE is based solely on indirect observations, and in particular labeling with antibodies raised against this epitope [78]. In a very recent study based on the SIV gp41, it was convincingly shown that the reported surface labeling was due to the release of Env from expressing cells into the supernatant; shed Env subsequently attaches to the surface of cells in the culture [78]. The authors report that transferring supernatant from Env-transfected cells to mock-transfected cells was sufficient for labeling of the mock-transfected cells. Furthermore, the PNG in the KE was never glycosylated, and both serum and monoclonal antibodies against the KE failed to neutralize even highly neutralization-sensitive strains [78].

#### LLP-2

Biochemical studies led to the suggestion that LLP-2 may also be a traversing peptide, and that it interacts with the extracellular 6-helix bundle at the moment of fusion [60] (Figure 2C). LLP-2 exposure may be induced by CD4 and CCR5 binding, and be highly transient, as implied by the observations that antibody binding is observed at 31.5°C but not at 37°C and that LLP-2 peptides bind peptides mimicking the 6-helix bundle in biochemical experiments [60]. The mechanisms underlying this crossing of the membrane are unclear; the exact extent of membrane crossing by LLP-2 has not been investigated, so this remains speculative. As this study was based on a biochemical approach using peptides and antibody binding, the possibility of a biased interpretation due to labeling of shed Env subsequently bound to the cell surface, as described by Postler et al. for KE epitopes [79], cannot be excluded. Furthermore, the absence of other viral proteins from the Env-transfected cell system pleads for caution in interpreting the results, as indicated by the authors themselves [60]. Furthermore, Steckbeck et al. described the introduction of known epitopes into LLP-2, and failed to detect any extracellular or extraviral labeling, whereas similar epitopes inserted within KE epitopes were successfully detected [71].

These diverse results are such that caution is still required for any conclusions concerning a cell-versus-virion conformation of Env. It remains unclear whether there are such transient rearrangements and biochemical adjustments of Env in response to modifications in the membrane lipid or protein environment during viral assembly or at the moment of fusion; the corresponding observations may be experimental artifacts that do not reflect to biological realities. No images are available of the conformation and distribution of Env gp41-CT in the immature forming virion or in the mature virion. If the gp41-CT does adopt different topologies and if both the KE and LLP-2 are indeed membrane-traversing peptides, it would be interesting to investigate whether both can be found extracellularly at the same time, or whether such a conformation would be too highly unfavorable.

#### Open questions

A series of questions remain unanswered. For example, why is there such strong constraint on the structure of the LLP peptides but not on their sequences? How does this structure favor infectivity, motility or clustering of the Env in the virion? Does the conformation of the gp41-CT change upon virion maturation p55^Gag^ cleavage by the viral protease? Does the gp41-CT really adopt different topologies? If so, what triggers the switch from an intraviral/intracellular to extraviral/extracellular localization of KE or of LLP-2, and how are the KE and LLP-2 retained intravirally or “freed”? And what would the function of transiently exposing the highly immunogenic KE be? Alternatively, if the immune response elicited by the KE is due to Env shedding rather than to extracellular exposure, could this be a viral strategy to evade the immune system, by providing immunodominant domains that are subsequently not accessible to antibodies as they are located inside the virion and infected cells?

### Viral assembly, Env incorporation into virions and viral infectivity

Env is produced in the RER and the Golgi network, whereas the p55^Gag^ and p160^GagPol^ protein precursors and the accessory proteins are synthesized by cytosolic ribosomes. Viral assembly thus requires viral components synthesized in distinct cellular compartments and the genomic RNA to come together and to interact at the budding interface. Viral assembly is a highly regulated phenomenon, dictated by intracellular trafficking.

**Figure 3:**
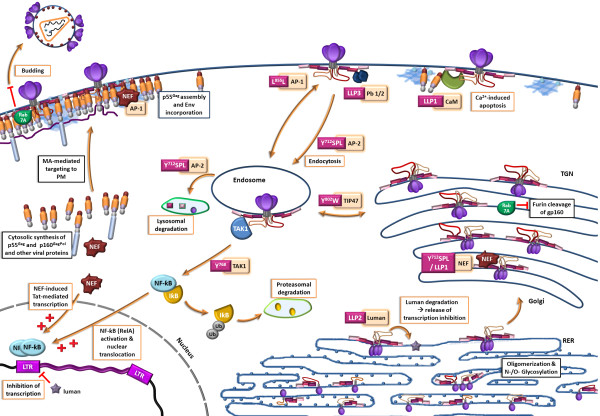
**Schematic representation of the interacting partners of the gp41-CT in the cell.** The Env precursor gp160 is synthesized in the rough endothelial reticulum (RER), where it is glycosylated and oligomerizes into trimers. Furin cleavage in the Golgi apparatus enables the formation of the gp120-gp41 structure. Once it reaches the plasma membrane (PM), Env is subject to endocytosis, via the Y_712_SPL and L_855_L_856_ motifs interacting with AP-1 and AP-2. Cycling through the trans Golgi network and between endosomes is ensured through interactions with TIP47, via the Y_802_W_803_ diaromatic domain, and with AP-1 via Y_712_SPL. Env is targeted to the PM through Y_712_SPL interaction with AP-1 and AP-3. The p55^Gag^ precursor is synthesized in the cytoplasm and accumulates at the PM in lipid raft-rich domains. Interaction of the gp41-CT (through LLP-2) with the Matrix protein (MA) ensures Env incorporation into the budding virion. gp41-CT interacts with Nef through L855L856. NF-κB activation through phosphorylation of IκB is mediated by interaction of the Y_768_ with TAK-1. Interference with luman-mediated inhibition of transcription is mediated by LLP-2. Abbreviations: RER: rough endoplasmic reticulum; TGN: trans Golgi network; CaM: calmodulin; LLP: lentiviral lytic peptide; TAK: TFG-β-activating factor; AP: adaptor protein: TIP47: Tail-interacting protein 47 kDa; Ub: ubiquitin ; Phb: Prohibitin.

HIV assembly and budding occurs at the plasma membrane (PM) [80-84] in detergent-resistant-membrane lipid raft-enriched domains [73,85-87] (Figure 3). The p55^Gag^ precursor is a polyprotein, including the structural proteins of HIV, namely the matrix protein (MA) lining the cell-derived viral envelope, the capsid protein (CA), constituting the viral cone-shaped capsid containing the viral RNA genome, the nucleocapsid (NC, p7), associated with the viral RNA genome, and p6, regulating the viral protease, as well as two spacer peptides, SP1 and SP2. MA drives and regulates p55^Gag^ targeting to the PM by preferentially interacting with phosphatidylinositol 4,5 bisphosphate [PI(4,5)P2] [88-90]. Once it has reached the PM, the p55^Gag^ precursor is anchored by its N-terminal myristoylated moiety [91,92] and stabilized by basic residues 84–88 [89,90,92]. The myristoylated moiety is co-translationally coupled to the p55^Gag^ MA domain, and its exposure is regulated by binding to calmodulin: binding to calmodulin induces conformational changes within MA [93,94] which lead to p55^Gag^ binding to lipid rafts [95-97], where it oligomerises [73,86,87,98], through the C-terminal domain of CA and the α-helical spacer peptide SP1, leading to the formation of the immature lattice [99,100]. In solution, MA within the p55^Gag^ precursor assembles in trimers [101]. In cholesterol-rich membranes composed of phosphatidylcholine, cholesterol and [PI(4,5)P2], myristoylated MA assembles in hexamers of trimers interconnecting the radially oriented immature p55^Gag^ lattice [99,102,103].

Four models of viral assembly and budding have been described exhaustively in three recent reviews [25,104,105] and will therefore not be addressed in detail again here. Briefly, the “Passive incorporation model” posits that HIV assembly occurs by random co-localization of p55^Gag^ and Env at the cell membrane; it is supported by the observations that a number of cellular membrane proteins are incorporated into virions without stringent restriction, that HIV-1 can be pseudotyped by a variety of retroviral and non-retroviral envelopes (*e.g.* alphaviruses, ortho/paramixoviruses, flaviviruses, etc.) [106-109], and by the fact that gp41-CT truncations do not affect Env incorporation in some cell types (*e.g.* the HeLa cell line) (reviewed in [25,104]). The three other models, in contrast, propose that p55^Gag^ and Env are co-targeted to assembly platforms in a highly regulated fashion, either through direct p55^Gag^/Env interaction, or through an indirect interaction mediated by lipid rafts, or by other cellular factors (for details, see [25]). Indeed, several lines of evidence suggest that p55^Gag^ and the gp41-CT interact [52,110-118]. However, whether the MA/gp41-CT interaction is direct or involves other partners remains controversial and requires further investigation: coimmunoprecipitation studies and cell-free protein-protein interaction studies suggest a direct interaction between gp41-CT and p55^Gag^[112,113,116], but further interactions involving cellular or other viral proteins, such as adaptins AP-1 through AP-4 or TIP47, lipid rafts themselves and other lipid raft components have also been described. One possible explanation for this diversity of reports is that the interacting partners may have stabilizing or facilitating effects, that are not required at all moments, or that are inessential in some cell types. The observation that gp41-CT truncation alters the localization and affects polarization of the viral capsid at the viral synapse (VS) in some cell types (see section ‘Phenotypes of gp41-CT truncations’) supports this view [119,120].

#### Interaction of the gp41-CT with Gag

In the absence of Env, Gag and viral-like particles (VLPs) bud in a non-polarized manner from the epithelial cell membrane suggesting the existence of a p55^Gag^/Env interaction. Env drives p55^Gag^ accumulation and virion budding from the basolateral surface of polarized cells (MDCK cells in early reports, then extended to all polarized cells) [111,121,122]. This polarized release was shown both to be mediated by the Y_712_SPL motif of the gp41-CT and to require interaction between Env and MA [111,121,122]. Likewise, truncation of the gp41-CT of a SIV strain resulted in redistribution of cell surface envelope molecules from localized patches to a diffuse pattern that covered the entire plasma membrane [123]. Conversely, Env glycoproteins produced alone are randomly scattered across the cell plasma membrane, whereas in the presence of p55^Gag^ they are recruited to budding sites, suggesting active recruitment to these sites [109].

Further evidence that a Gag/Env interaction is required for the process of viral assembly arises from the observation that although truncation of the entire gp41-CT generally has little or no effect on Env incorporation, and even increases Env expression at the cell membrane, deletions downstream of the KE (ablating the LLP domains) or small deletions within LLP-2, severely hamper Env incorporation [43-46,52]. This defect can be reversed by mutations (V34I) in MA [52]. Conversely, it was reported that mutations in the p55^Gag^ precursor MA protein result in decreased plasma membrane expression of Env [110], and in lower Env stability and virion incorporation [110,119,124,125]. The discovery that mutations in MA interfere with Env incorporation in ‘non-permissive’ cells and that pseudotyping with shorter Envs (MLV or a truncated HIV-2) or truncating the HIV-1 gp41-CT reversed this impairment confirm that the gp41-CT interacts with the p55^Gag^ precursor [52,114,115,118]. Importantly, these findings have led to the idea that the gp41-CT plays a role in assembly during the late stages of the viral cycle, rather than in Env fusogenicity and in entry during the early stages of the replication cycle [52,114,118].

The MA/Env interaction has variously been mapped to L12 and L30 within MA [52,114,118], to K32 [110], and to the MA-CA junction [114-116,118]. The defect in Env incorporation into virions containing the L12E mutation in MA can be reversed by truncations of the gp41-CT depriving it of the LLP domains [114,115,118], and MA AA L30 has been shown to interact with the C-terminus of LLP-2 [52]. Other studies based on progressive HIV-1 or SIV gp41-CT truncation mutants (67 AA, or a central peptide of 93 AA) confirmed that this interaction mapped to LLP-2 [52,112,113,126]. Another group however repeatedly mapped the interaction to the 28 C-terminal AA (i.e. to LLP-1) [47,54,116]. Despite the diversity of truncations that have been studied, the observation that removal of 59 to 90 AA from the gp41-CT strongly impairs Env incorporation into virions whereas removing 93 AA from the C-terminus does not affect Env incorporation [115,118] suggests that LLP-2 does not adopt an appropriate conformation in the absence of LLP-1, and fails to accommodate the p55^Gag^ precursor. Lastly, Env and MA sequences from patient-derived viral strains co-evolved over time [127], providing further *ex vivo* support for a gp41-CT/p55^Gag^ interaction.

The gp41-CT/p55^Gag^ interaction seems to tolerate a degree of flexibility, as MA mutations that abolish incorporation of HIV-1 Env do not interfere with the incorporation of heterologous retroviral Envs with short CTs [115,118] and as HIV can be pseudotyped with a variety of retroviral and non-retroviral Env glycoproteins [106-109]. This contrasts with VSV cores, which cannot be pseudotyped by HIV gp160 [128]. The Env/Env interaction mediated by the gp41-CT [33], in contrast, is highly specific as recent studies mixing Env glycoproteins from lentiviruses (HIV or SIV) and from two retroviruses (RSV and MLV) to produce pseudovirions show that Env clustering at the plasma membrane is dependent on the Env gp41-CT and is limited to autologous Envs [106,109]; also in another study, the formation of hybrid pseudovirions was not tolerated, although in this case, the non-retroviral Ebola Env glycoprotein and HIV were mixed [108].

#### Viral stiffness, viral maturation and infectivity

It is currently believed that the p55^Gag^ precursor orchestrates viral assembly through interactions with all viral components to be incorporated, and that the gp41-CT/p55^Gag^ interaction is involved in controlling viral infectivity in a maturation-dependent fashion. The efficiency of entry and fusion is lower for immature than mature HIV-1 particles [47,54,129]. Similar results have been reported for the SIV Env-CT [117]. This inefficiency can be overcome by truncation of the gp41-CT or by LLP-1 truncations [47,54,114].

It has been proposed that the plasticity of the lentiviral particle controls its infectivity and fusion capacity [32,130]. Immature virions are 14 times stiffer than mature particles [32]. The structural rearrangements associated with p55^Gag^ maturation by the viral protease transform the stiff immature viral particle into a mature particle with a thin protein shell and conical core; its ability to enter cells depends on its softness [32,130]. p55^Gag^ maturation by the viral protease into its distinct components is a regulated and sequential process with particular kinetics [131]: the first cleavage is that of the SP1-NC, and it induces the structural switch of Gag that controls maturation and activates Env [54,99]. STED microscopy has recently provided visual evidence of such regulation: Env spikes at the surface of mature virions coalesce into foci, whereas in the less infectious immature virion, Env spikes remain scattered across the virion surface [33]. Proteolytic maturation of p55^Gag^ allows Env lateral motility and clustering into foci [33,132]. However, although truncation of the gp41-CT also favors Env motility on immature particles, it does not result in Env clustering: this provides evidence that gp41-CT-mediated Env clustering optimizes viral infectivity [33,132], confirming prior reports showing that LLP-1 and LLP-2 mediate Env clustering [55], partly via Arg residues [56]. Pang et al. [32] recently used atomic force microscopy to demonstrate a correlation between viral infectivity and virion stiffness. Using different constructs that separate the Env ‘fusion-activity’ (Env∆CT construct) from the ‘stiffness/softness properties’ (GFP-gp41-CT construct), they found that the gp41-CT stiffens immature virions in a concentration-dependent fashion, thereby impairing infectivity of immature (but not mature) particles [32]. Because VSV-G pseudovirions were similarly affected, the authors conclude that particle stiffness directly regulates immature viral entry [32]. Intriguingly, there was no direct interaction between the Env∆CT and the GFP-gp41-CT constructs, implying that the gp41-CT directly stiffens immature viral particles, and that virion stiffness controls virion fusion capacity [32]. These studies indicate biophysical mechanisms underlying the control of viral infectivity by protease-mediated p55^Gag^ cleavage [32,33,132]. Although both groups related viral infectivity to viral stiffness, they draw opposite conclusions about the roles of viral stiffness and of the gp41-CT: Chojnacki et al. [33] propose that the stiffness of the immature particle interferes with Env motility and clustering through the gp41-CT, whereas Pang et al. [32] propose that it is viral stiffness itself that is controlled by the gp41-CT through an as yet undefined mechanism possibly involving the arrangement or stability of the immature Gag shell. In other words, stiffness impairs Env motility and gp41-CT-mediated Env clustering and infectivity [33,132], or, alternatively, stiffness is a consequence of the presence of the gp41-CT [32]. Either way, Env ensures minimal fusion until the virus is mature, suggesting a mechanism whereby unprocessed p55^Gag^ “withholds” Env until full maturation by the viral protease is complete through an inside-out regulation mechanism [54,58,129].

#### Control of viral infectivity

The p55^Gag^ precursor is trafficked to and accumulates at the plasma membrane where it recruits viral components (including Env, viral genomic RNA, viral accessory proteins). However, interactions of the gp41-CT with cellular trafficking regulators (discussed below, ‘Interactions of the gp41-CT with cellular factors and intracellular regulators’) down-modulates Env as soon as it reaches the plasma membrane such that it remains scarce until p55^Gag^ oligomerization is complete or, at least, sufficiently dense in the assembly platforms to ensure efficient viral assembly and release [133] (Figure 3). This mechanism for the regulation of the surface abundance of Env protein may protect infected cells from Env-dependent cytopathic effects, from Env-induced Fas-mediated apoptosis and/or from Env-specific immune responses [133-137]. During, or shortly after virus release, the viral protease cleaves the p55^Gag^ and p160^GagPol^ precursors, resulting in structural and morphological rearrangements of the virion that enable Env fusion capacity [47,54,129]. gp41-CT interacts with p55^Gag^ with higher affinity than with mature MA [129], and this loosened interaction probably favors changes in Env conformation after viral maturation [47,54] and Env lateral motility [33]. With the completion of maturation, the virion becomes maximally infectious and can start a new round of infection. The scarcity of Env spikes at the surface of virions appears to be a compromise between efficient fusion capacity, through virion softness, clustering of Env into foci, and immune escape.

This mechanism for controlling infectivity differs from that of other retroviruses, such as Murine Leukemia Virus and Mason-Pfizer monkey virus, where the viral gp41-CT is cleaved by the viral protease in the released virion, resulting in Env becoming fusion competent [138,139].

#### Open questions

Clearly, p55^Gag^ is the main pillar of HIV assembly, and recruits the constituents of HIV virions, orchestrates their assembly and controls infectivity of viral particles. Nevertheless, many questions concerning Env recruitment and incorporation into virions remain unresolved. Do p55^Gag^ and Env interact directly or is this interaction indirect? Or, alternatively, are there many contact points, both direct and indirect? In this case, are these multiple interactions permanent and simultaneous, or is there a sequential order important for the control of viral assembly and/or infectivity? Which Env determinants are involved? Many cellular proteins have been described to interact with the gp41-CT (detailed below, section ‘Interactions of the gp41-CT with cellular factors and intracellular regulators’), but it is not known which bridge the p55^Gag^/Env interaction and which serve other purposes. It is unclear which are, as a consequence, incorporated in newly synthesized viral particle, and if they are, whether they are included passively or contribute to the initial steps of the following infection round.

### Phenotypes of gp41-CT truncations

Despite numerous studies addressing the role of the gp41-CT domain, the roles of particular determinants remain poorly documented. One reason why the gp41-CT remains poorly understood is that the effects of gp41-CT truncations and/or mutations are cell-type dependent. For example, many transformed T-cell lines (*e.g.* the cell lines H9, MT-2, Jurkat, and CEMx174) and primary target cells of HIV (primary CD4+ T cells and macrophages) do not sustain replicative spread of viruses with a truncated gp41-CT; these cells are referred to as ‘non-permissive’ for gp41-CT truncation. Other cells, in contrast, such as the T cell lines MT-4 and C8166 and several laboratory cell lines (HEK293T, HeLa, and COS) are less affected by full or partial truncations of the gp41-CT and sustain replication of the corresponding viruses (termed ‘permissive’ cells) [43-45,119,120,140-142]. Truncation of gp41-CT decreases Env incorporation into virions by about 10-fold in ‘non-permissive’ cell lines and in primary HIV target cells (i.e. primary PBMCs and monocyte-derived-macrophages), but has a much smaller effect in ‘permissive cells’ [119,120]. An illustrative example, albeit extreme, is that truncation mutants have different effects in macrophages [119], perhaps owing to differentiation and culture conditions. Compromised infectivity and replication capacity in ‘non-permissive’ cells has been linked to altered localization of the viral capsid that failed to accumulate and polarize at the viral synapse [119,120], but not to increased gp120 shedding from virions nor to reduced Env cell-surface expression [110,119,120,140]. HIV and SIV viral particles contain relatively small amounts of Env (~7-14 Env trimers/virion) [143-145], due to both the massive recycling of neo-synthesized Env and to Env shedding. These findings and comparisons are thus difficult to interpret and their biological relevance is unclear.

It is not clear how gp41-CT truncations may impact Env incorporation into virions without affecting Env expression at the PM or Env trafficking through the ER and Golgi networks, as one could expect Env incorporation into virions to be related to the amount of Env at the PM. One possible explanation is that inherent differences between cell types in the expression or localization of host factors play a role in viral assembly; this would account for the cell-type dependent phenotypes of gp41-CT truncations and mutations. A second possible explanation is that because the gp41-CT activates the NF-κB canonical pathway [79], gp41-CT truncations interfere with the cell activation status (see section ‘Activation of transcription’). Cell types and cell lines differ in their basal activation levels and in their dependence on NF-κB, and it is therefore conceivable that truncations of the gp41-CT could translate into cell-type-dependent phenotypes [31].

One recent study [141] compared the infectivity of sub-saturating titers of wild-type and of a HIV-1 gp41-CT-truncated mutant in various gp41-CT truncation ‘permissive’ (MT-4, C8166) and ‘non-permissive’ (H9, MT-2, Jurkat, CEM-SS) cell lines: the ‘permissive’ and ‘non-permissive’ phenotypes of target cells correlated with the level of gene expression, and particularly of p55^Gag^ production and processing to mature capsid (CA), rather than with Env incorporation into the virion [141]. In ‘non-permissive’ cells, viral assembly was compromised as a consequence of p55^Gag^ failing to accumulate and polarize at the viral synapse (VS), whereas in ‘permissive’ cell lines, high levels of p55^Gag^ overrode and compensated for the absence of the gp41-CT [141]. This study could reconcile the cell-type dependence of Env incorporation with a dual role for the gp41-CT, being involved in Env incorporation into virions and polarized budding on one hand and in cell activation and regulating viral gene transcription on the other.

The situation is more clear-cut for the SIV gp41-CT. SIV Env truncations have been reported to arise spontaneously when culturing SIV in human cell lines, and then to revert back following culture in simian cells [146-149]. These truncations increased Env surface expression, fusogenicity [61,150] and infectivity *in vitro*[147,151]. These SIV gp41-CT truncation mutants are replication competent both *in vitro* and *in vivo*, but lack full pathogenicity *in vivo:* macaques infected with a LLP-1- and LLP-2-defective SIVmac239 initially had viremia, but the viral load declined with time to below the detection threshold; the infected macaques never progressed to AIDS [152]. A similar phenotype has been reported for HIV-2 [147,153]. Spontaneous truncations of the gp41-CT of SIV cultured in human cell lines and reversion in rhesus PBMCs may reflect the presence of a restriction factor whose action is inhibited by the gp41-CT in rhesus cells, but which is absent from human PBMCs or ineffective against SIV [79]. Alternatively, spontaneous truncations of the gp41-CT in human PBMCs or T cell lines may improve viral infectivity by enhancing Env expression and thereby incorporation into virions. The truncation of the SIV gp41-CT observed in human cell lines may remove signals mediating Env trafficking such that the higher Env density at the surface of SIV virions is preserved. Further studies to elucidate how these phenomena are related and the real consequences of Env levels in the virion may reveal new leads for the development of strategies to control viral assembly.

### Interactions of the gp41-CT with cellular factors and intracellular regulators

A number of cell factors have been described to interact with the gp41-CT: in some cases, the interactions have simply been described without any evidence of significance, and in others, the significance is controversial. Nonetheless, most of these interactions between the gp41-CT and cellular factors contribute to improving viral replication, by promoting viral gene expression, enhancing viral infectivity, or interfering with intrinsic cellular restriction factors or immunity. Innate immunity has developed many (often interferon-induced) pathways to counter gene expression and the late stages of the viral replication cycle, namely assembly and budding, as these are essential steps for viral propagation. It seems likely that such factors are, at least in part, responsible for the cell-type-dependent phenotypes observed.

**Figure 4:**
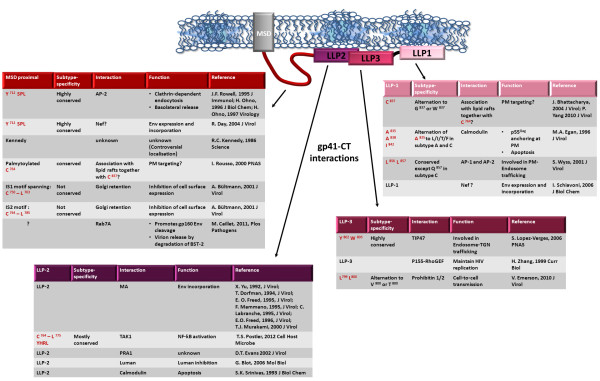
**gp41-CT domains or AA with reported function(s), conservation across subtypes, interacting partners and role of these interactions.** Abbreviations: RER: rough endoplasmic reticulum; TGN: trans Golgi network; CaM: calmodulin; LLP: lentiviral lytic peptide; TAK: TFG-β-activating factor; AP: adaptor protein: TIP-47: Tail-interacting protein 47 kDa; Ub: ubiquitin.

Figure 3 and 4 summarize cellular and viral proteins interacting with the gp41-CT and their intracellular localization.

#### Env trafficking and sorting through the trans Golgi network (TGN)

Golgi retrieval signals function to return proteins which have left the Golgi to that compartment; Golgi retention signals, by contrast, are generally found in the transmembrane domain of Golgi and ER resident proteins. The main Golgi retrieval signals are cytoplasmic domain tyrosine-based or dileucine motifs [154,155]. The gp41-CT contains both Golgi retrieval (tyrosine-based and dileucine-based) and Golgi retention motifs [156]. They regulate Env trafficking through the endocytic pathway [157-159].

#### AP-mediated Env endocytosis

As soon as Env comes into contact with the plasma membrane, it is endocytosed. Time course analyses show that 50% of Env, but only 15% of a gp41-CT-truncated Env, is internalized after 60 minutes [157]; and similar time courses have been reported using a chimeric CD4 molecule fused to the gp41-CT [156]. These observations indicate that the gp41-CT mediates endocytosis. Env is endocytosed via interactions with the clathrin adaptor complex (AP proteins) [157-159]. Clathrin adaptors sort and direct proteins through the endocytic pathway. The highly conserved Y_712_SPL motif of HIV (and Y_721_XXΦ of SIV) immediately downstream from the transmembrane region has been implicated in regulating Env intracellular trafficking by binding clathrin adaptor AP-2 μ (medium) chains [157-159] (Figure 3 and 4). The Y_712_SPL motif can also interact with the AP-1 and AP-3 adaptor complexes through μ1 and μ3A, respectively [159]. Whereas the interaction of the gp41-CT Y_712_SPL motif with AP-2 leads to Env internalization, interaction with AP-1 regulates Env trafficking through the TGN and addresses Env to the basolateral PM in polarized cells [159] (Figure 3 and 4). The AP-2 adaptor complex also mediates p55^Gag^ internalization through Y_132_ and V_135_ at the MA-CA junction [160], and the AP-1 μ [161] and AP-3 β and δ subunits contribute to p55^Gag^ release. They do so by transporting it to intracellular sites of active budding, such as MVBs and the PM [162,163] and/or by facilitating its interaction with other cellular partners such as Tsg101 [161]. The interaction between Env and various proteins of the AP clathrin adaptor complex may therefore be involved in the p55^Gag^/gp41-CT interaction and determine the fate of Env (targeting to the lysosomal compartment for degradation, or to the basolateral plasma membrane for incorporation into virions).

Y_712_SPL-mediated endocytosis is suppressed in the presence of the HIV-1 p55^Gag^ precursor polyprotein [133,164]. This suggests that p55^Gag^ interferes with the Env/AP-2 interaction and that the amount of Env at the cell surface is regulated by the AP-2 versus p55^Gag^ balance. AP-2 may thus eliminate any Env protein that is not associated with p55^Gag^ and that is therefore not destined for incorporation into virions [133,164]. Env internalization may proceed until sufficient p55^Gag^ has been synthesized and has accumulated at the site of viral assembly, at which point, p55^Gag^ would interact with Env directly or indirectly, thereby trapping Env into the nascent virion [133,165]. In the case of SIV, mutating the Y_721_ in agp41-CT truncated Env resulted in increased Env expression at the plasma membrane, whereas mutations of Y_721_ in a full-length gp41-CT context had little impact on Env expression at the plasma membrane [123,166]. Presumably, other motifs involved in endocytic regulation overcome the single mutation.

Additional distal determinants also regulate Env internalization [164]. These have now been identified as being the most distal C-terminal dileucine motif [167,168] and/or IS1 and IS2 Golgi retention sequences [156]. Recruitment of AP-1 [167] and AP-2 [168] by the most C-terminal dileucine motif ensures correct post-Golgi routing of Env, and restricts cell surface expression of Env in conjunction with the membrane-proximal Y_712_SPL motif [167,168] (Figure 3 and 4).

Both the Y_712_SPL motif and the most C-terminal dileucine motif are extremely well conserved across HIV-1 subtypes (except subtype C), and also in HIV-2 and SIV strains, evidence of their importance (Figure 1B). Similar signals in cellular proteins or in the glycoproteins of other viruses (*e.g.* alpha herpesviruses) affect endocytosis as well as intracellular trafficking in endosomes and the TGN, and can determine basolateral versus apical sorting (reviewed in [169,170]). It is nevertheless intriguing that subtype C viruses harbor a LQ rather than LL (dileucine) at this position in over 60% of cases. As the requirement for an intact C-terminal dileucine motif for correct interaction with AP-1 and with Nef (see below, section ‘Assembly’) seems to be quite stringent [167], these observations raise three possibilities: (i) other motifs are involved, or (ii) Env trafficking differs between subtypes, and differences in sub-cellular localization are less detrimental to viral assembly and infectivity than previously believed, or (iii) subtype C Env internalization is less efficient and more Env is expressed at the surface of infected cells and possibly on virions of subtype C than of other subtypes; this could be a cause or consequence of the weaker antibody response or of the lower replicative capacity.

Another tyrosine-based motif, Y_768_HRL, is present in the gp41-CT. Although this motif also interacts with AP-1, AP-2 and AP-3, it is not involved in endocytosis of Env, perhaps because of its localization in the gp41-CT, but may mediate NF-κB gene expression [79] (see below, section ‘Activation of Transcription’). Likewise, one or two other YxxΦ motifs are present in various SIV strains and in HIV-2, but none has been shown to mediate endocytosis [166].

#### Golgi retention

Most Env is retained in the ER, and from there ubiquitinated and degraded by the proteasome. Only a small proportion actually reaches the Golgi and proceeds to the cell surface. Nevertheless, Env is also retained in the Golgi [156]. The IS1 (AA 750–763) and IS2 (AA 764–785) regions in the gp41-CT reduce Env surface expression by retaining Env in the Golgi, independently of AP-1 and AP-2 [156] (Figure 4). These sequences include LL (or IL motifs, which behave similarly to LL in Golgi retrieval [171]), and result in retention in the Golgi of gp160 and of chimeric proteins composed of the gp41-CT fused to the CD4 receptor or to immunoglobulin extracellular/transmembrane domains. These sequences are also highly conserved across subtypes [156] (Figure 1B).

#### TIP47

The leucine-zipper domain, LLP-3, also contains a highly conserved Y_802_W_803_ diaromatic motif. This motif has been found to be involved in the retrograde transport of Mannose-6-Phosphate Receptors and in lipid droplet biogenesis. The Y_802_W_803_ motif in Env ensures the retrograde transport of Env from late endosomes to the TGN (Figure 3) and thereby contributes to the regulation of the intracellular distribution of Env, and to Env incorporation into the virion, particularly in macrophages [165,172-175]. Lopez-Vergèz et al. reported that TIP47 is required for the p55^Gag^/gp41-CT interaction [165]. Disruption of the p55^Gag^/TIP47 interaction through mutations in MA or by TIP47 knock-down causes p55^Gag^ to localize in scattered dots in the vicinity of the PM. These observations led to the suggestion that TIP47 acts as a linker between Env and p55^Gag^, and serves as a bridge for efficient Env incorporation [175]. However, other authors report not being able to reproduce these findings [25].

#### Rab7A and other RabGTPases

Rab proteins are early and late-endosome associated GTPases that regulate specific steps in intracellular membrane trafficking. They recruit tethering, docking and fusion factors, as well as the actin- and microtubule-based motor proteins that facilitate vesicle traffic [176-180]. In a study aiming to identify cellular trafficking factors involved in HIV assembly and budding, Caillet et al. report that Rab4A, Rab6A, Rab8A, Rab9A, and Rab11A moderately decreased viral infection (by 30-60%) in HeLa cells, probably in relation to their roles as regulators of the exocytic pathway [173,181,182]. Rab7A knock-down resulted in a?>?30-fold decrease in viral propagation [182]. Rab7A is involved in regulating exit from the late endosome/MVB by controlling the maturation and fusion of late endosomes and phagosomes with lysosomes, their intracellular motility when loaded with lipids, and their interaction with the cytoskeleton for migration along microtubules [176,178-180,183-185]. Of particular interest is the finding that Rab7A has a dual activity. Rab7A knock-down experiments using siRNA also showed that Rab7A regulated and allowed gp160 processing to gp120?+?gp41 and incorporation of mature Env into particles (Figure 3). The authors propose that Rab7A may either ensure correct Env trafficking through furin-containing compartments, or ensure appropriate co-localization of Env and/or of furin; alternatively, Rab7A may modulate Env expression, and in the absence of Rab7A, Env is expressed to levels that exceed the processing capacity of furin [182].

Rab7A knock-down also interfered with viral release, resulting in the accumulation of gp160 and viral components at the PM, reminiscent of a BST-2/Tetherin phenotype [182]. The authors report that Rab7A is required for sorting BST-2/Tetherin to lysosomes for degradation through molecular mechanisms that remain to be elucidated [182] (Figure 3). Rab7A is also involved in the late maturation of the final autophagic vacuoles [186,187].

Strikingly, the role of Rab7A shares similarities with that of Nef (see section ‘Nef’ below) in mediating increased Env at the PM [188] and in intercepting the autophagic processing of Gag to increase virion assembly and egress [189,190]; both also affect traffic through the endocytic pathway [106]. It would be interesting to find out whether Nef intersects the Rab7A or Rab5 signaling cascades.

Rab9A is a mediator of late endosome-to-TGN trafficking involved in the replication of many viruses, including Marburg Virus, Filoviruses and Measles. It interacts with TIP47 and PRA1 (prenylated Rab acceptor 1), both cellular interacting partners of gp41-CT [173]. PRA1 has been reported to interact with lentiviral gp41-CT (SIV, HIV-1 and 2, EIAV and FIV) in a 2-hybrid system [191] without modulating viral release, infectivity or Env incorporation [192]. The significance of these interactions remains unclear, but as both Rab9A and PRA-1 are involved in vesicular trafficking, it is possible that they participate in regulating Env trafficking through the Golgi.

### Assembly

#### Detergent-resistant lipid rafts

Detergent-resistant lipid rafts are cellular microdomains enriched in cholesterol and sphingolipids and which contain proteins. Lipid rafts are thought to play an important role in many cellular processes including signal transduction, membrane trafficking, protein oligomerization, cytoskeletal organization, and pathogen entry (reviewed in [193-195]). Lipid rafts are thought to be central to the assembly and infectivity of numerous enveloped viruses, including lentiviruses. The lipid bilayer of HIV virions is highly enriched in cholesterol and sphingomyelin, both of which are major components of lipid rafts [196,197]. Also, p55^Gag^ and Env and have been co-localized with lipid raft-associated proteins [73,85-87]. Env incorporation into lipid rafts optimizes its incorporation into virions and confers viral infectivity [198]. Conversely, excluding HIV from lipid rafts impairs viral infectivity [85,199-203]. The 12 C-terminal amino acids of LLP-1 and portions of LLP-3 [43,44,49,204], and also palmitoylated cysteines C_764_ and C_837_, and bulky hydrophobic side chains, have been implicated in targeting Env to lipid rafts [198,205] (Figure 4). In contrast to the reports by Bhattacharya [198,206], Chan et al. [207] found that Cys to Ser and Cys to Ala mutations had no significant effect on Env expression, trafficking, interaction with lipid rafts and incorporation into virions, or on the fusion efficiency or infectivity in single-cycle infections, despite decreased palmytoylation of Env [207].

Other studies argue against any substantial direct targeting of Env to lipid rafts, and suggest, rather, that it is p55^Gag^ that recruits and anchors Env to lipid rafts [85]. Various observations imply that p55^Gag^ drives Env to lipid rafts. First, mutation of residue S4 in MA abolishes the interaction of p55^Gag^ with lipid rafts resulting in reduced Env incorporation into virions, presumably by inhibiting the ability of p55^Gag^ to target or hold Env within raft microdomains [208]. Second, independent studies have reported that in the absence of p55^Gag^, or in the presence of MA or gp41-CT mutations that abolish Env incorporation into virions, Env was not found associated with detergent-resistant membranes [198,206]. The observation that neither the cysteine residue in LLP-1 nor the presence of bulky AA is conserved between subtypes raises further doubts about a direct interaction between Env and lipid rafts. This polymorphic flexibility is more consistent with the view that Env is recruited to lipid rafts through p55^Gag^, and/or that other determinants are also involved. Nonetheless, it cannot be excluded that under certain circumstances, particularly in transfection experiments involving strong viral gene overexpression, Env may be passively incorporated into virions; a phenomenon of this type would explain the contrasting results reported.

In the cases of other enveloped viruses, such as Influenza Virus [209] and Respiratory Syncytial Virus [210], the matrix/lipid raft interaction is favored by, or even dependent on, the presence of the respective envelope cytoplasmic tails. Whether lentiviral gp41-CTs also somehow influence the MA/lipid raft interaction has not been described.

#### Nef

Interplay between the Env gp41-CT and the viral accessory protein Nef has been reported to increase viral infectivity [211,212]. The viral accessory protein Nef is a pleiotropic non-enzymatic, myristoylated, HIV and SIV lentivirus pathogenesis factor. Nef increases viral infectivity through various, not fully elucidated, mechanisms involving immune escape [213-216] (reviewed in [217]), increased viral infectivity and propagation [106,212,218-222], and perturbed immune activation, thereby promoting a favorable environment for viral replication [90,216,219,223-227]). Most of Nef’s functions are due to its ability to influence the cellular environment, mainly through protein-protein interactions which interfere with cell-receptor trafficking or are involved in T cell receptor (TCR)-induced signaling [228].

It has been proposed that Nef promotes Env gp160 precursor processing and PM expression of the mature gp120 and gp41 products by abrogating their retention in the cis-Golgi [106,212], and by specifically augmenting Env accumulation at the PM [212]. Enhanced Env at the PM was found to be dependent on the C-terminal dileucine motif of the gp41-CT and on the C-terminal residues of Nef (positions 181–210), and it was suggested that Nef increases Env incorporation into virions [212]. Whether the gp41-CT Golgi retention sequences IS1 and IS2 are also involved has not been investigated.

Others have proposed that Nef may modulate cell signaling thereby altering intracellular trafficking and augmenting Env accumulation in late endosomes/MVBs [106]. Nef-insensitive Env glycoproteins from various enveloped viruses share pH-dependence (Influenza-HA, VSV-G and HCV), and most importantly, lack endocytic traffic motifs, budding in compartments other than MVBs (HCV) or at the cell PM (Influenza-HA, VSV-G). Glycoproteins needing Nef for optimal assembly harbor determinants of intracellular trafficking through the endocytic pathway and assemble in MVBs [106,229]. Furthermore, the role of Nef in Env and p55^Gag^ precursor co-localization could be linked, at least to some extent, to Gag expression levels, and this may explain the cell-dependence and the gp41-CT ‘permissive’ versus ‘non-permissive’ phenotypes. The incorporation into pseudovirions of some retroviral and lentiviral Envs is increased in the presence of Nef, but not for those for which Env incorporation was already optimal [106]. Sandrin et al. conclude from these observations that Nef expression optimizes co-localization of retroviral Envs with p55^Gag^ in late endosomes/MVBs specifically for those Envs that inherently do not accumulate in the endosomal pathway [106]. This is consistent with the observation that Nef [231-233], the gp41-CT [167,168] and p55^Gag^[160-163] all interact with adaptins AP-1 through 3, suggesting these intermediates may be involved. It would be interesting to assess whether this observation is relevant to the findings of another study: the absence of the scaffold protein Dlg1 was described to restrict late steps of the HIV cycle due to Gag and Env rerouting and sequestration in a tetraspannin-rich (CD63+ and CD82+) late-endosome and plasma-membrane-related compartment [230].

Nef also interacts directly with the p55^Gag^ and p160^GagPol^ precursors via p6* in the overlap region between p6 and *pol*[234,235], and increases p55^Gag^ and p160^GagPol^ processing and egress via AIP-1/ALIX, a critical intermediate in the formation and proliferation of multivesicular bodies (MVBs) [221,233,234]. It has been suggested that the Nef-induced p55^Gag^ accumulation in late endosomes/MVBs is linked to autophagy. Autophagy is a cell survival and host defense mechanism by which cells degrade their own cytoplasmic constituents during starvation and microbial invasion; it involves clearing damaged organelles, macromolecular aggregates and intracellular microbes through double-membrane vacuoles, termed autophagosomes, which typically mature into amphisomes or autolysosomes after fusing with MVBs or lysosomes [236-238]. Nef has been suggested to increase virion assembly and egress by intercepting the autophagy process [189,190]; however, the mechanisms of HIV p55^Gag^ incorporation into the autophagosome leading to the release of HIV Gag particles into the extracellular environment remain largely unknown.

It is important to keep in mind, however, that recent work to identify the localization of HIV assembly in different cell types, and particularly in 293T cells and in macrophages, were performed using viral-like particles (VLPs) and VSV-G pseudotypes which target the p55^Gag^ precursor to the PM. From there, it either buds releasing new infectious viral particles or is endocytosed and accumulates in late endosomes and MVBs [80,81,239]. Most of these studies are based on the use of VLP and of pseudovirions comprising a heterologous Env, and on large p55^Gag^ or VLP/pseudovirion input, i.e. high Gag overexpression. The precise fate of p55^Gag^ precursor protein that has not budded from the PM and that has been endocytosed into late endosomes and MVBs is not known; more importantly, it remains to be established how and where Env and Nef interplay with the p55^Gag^ precursor to allow the release of fully infectious virions.

The Nef/gp41 interplay also involves other mechanisms independent of the gp41-CT and of its trafficking domains. Another function of Nef is to promote the biogenesis of viral assembly platforms including rafts and MVBs [221,222]. Nef can accumulate in lipid rafts and thereby contribute to infectivity of progeny virions by increasing the incorporation of lipids into viral membranes [222]. Whether Nef further favors or stabilizes the insufficient interaction of Env with lipid rafts, in conjunction or not with Gag, is still not known. In another study, the N-terminal region of Nef and its myristoylated moiety was shown to decrease the sensitivity of the gp41 ectodomain to neutralization by antibodies targeting the MPER epitopes that are embedded in the virion lipid bilayer (2F5 and 4E10) [240-242] and thereby contribute to viral escape from the humoral immune response [243]. This activity did not affect Env incorporation into virions, and the authors suggest that the susceptibility of neutralization to these epitopes may be modulated by Nef-mediated alteration of the lipid bilayer composition [243].

The consequences of Nef deletions, like those of gp41-CT truncations, are cell-type dependent [244,245]. In ‘Nef non-permissive’ cells, Nef deletion affects the early stages of viral infection and only slightly modulates the late stages of the viral replication cycle (assembly, release and maturation) [244,245]. Although there has been no direct pair-wise comparative study of gp41-CT truncations and Nef deletion mutants to our knowledge, it is interesting that there is some overlap between the gp41-CT and Nef ‘permissive’/’non-permissive’ phenotypes (*e.g.* MT-4 cells are ‘permissive’ to both, whereas primary CD4 cells and macrophages, and H9, Jurkat and CEMx174 cells are ‘non-permissive’ to both [119,120,244,245]). The reported effects of gp41-CT truncations and of Nef deletions on viral replication can differ in a given ‘non-permissive’ cell type (*e.g.* C1866 cells are ‘permissive’ to gp41-CT truncations [119,120] but are partially ‘non-permissive’ to Nef truncations) [244,245], although such comparisons should be interpreted with caution as the results come from different groups with different experimental set-ups. Day et al. reported that the incorporation of an Env Y_712_A mutant into virions was impaired in the absence of Nef in MT-4 cells but not in HEK293 cells [211]. This is intriguing because MT-4 cells are ‘permissive’ to both gp41-CT truncation and Nef deletion. One would have expected the Env Y712A ∆Nef double mutant not to display impaired Env incorporation when produced in this cell type. One possible explanation is that in gp41-CT ‘permissive’ cells, Nef plays a “compensatory” role: in the absence of Nef, the gp41-CT ‘permissive’ cell-line becomes ‘non-permissive’ to gp41-CT mutations. Alternatively, the correct routing of Env and/or Nef to intracellular trafficking compartments may play a crucial role in viral assembly and Env incorporation. Mutations disrupting the Y_712_SPL endocytic motif and truncation mutants may behave differently.

#### Calmodulin

Calmodulin is a calcium-induced mediator of FAS-mediated apoptosis. The gp41-CT binds calmodulin [246,247] and induces apoptosis of T cells, thereby accounting for most of the CD4+ T cell decline that characterizes HIV infection [134,248]. Amino acids Ala_835_, Ala_838_ and Ile_842_ of LLP-1 have been identified as being involved in calmodulin binding and inducing Ca^2+^ flux and DNA fragmentation in transfected cells [134,136,137,246,247] (Figure 4). Point mutations of the gp41-CT (A835W, A838W, A838I, and I842R) eliminate co-immunoprecipitation of Env with calmodulin but have no significant effect on viral production or Env expression [135,137]. Therefore, active Env cycling through the endocytic pathway may prevent Env-mediated apoptosis and thereby avoid premature cell-death and ensure viral assembly [133,164]. Because the gp41-CT/calmodulin interaction is deleterious to the virus by itself, it is likely that this interaction is “collateral damage” from other actions of calmodulin in viral assembly. These other actions include ensuring the conformational changes of MA so as to expose the myristoylated moiety that docks p55^Gag^ in assembly platforms at the PM [73,86,87,93,94,98].

Although independent reports have described Ala_835_ as being the calmodulin-binding site [136,137], Ala_835_ is not conserved across clades, and there is a Leu, Ile, Thr or Phe at the corresponding position in some strains. Possibly, polymorphism at this position explains the diverse calmodulin-binding activity of Envs and correlates with their apoptotic potential; indeed, the A835W mutation used in many studies fully abolishes the ability of the gp41-CT to bind calmodulin. If this were the case, late, more cytopathic or apoptosis-inducing Envs would be expected to all have an Ala_835_ and to bind more calmodulin, whereas the less pathogenic, early and chronic-infection viral isolates would be expected to bind less calmodulin and be more polymorphic at this position.

#### Prohibitin1/2

The dileucine motifs of LLP-2/LLP-3 (AA 790–800), and more specifically the dileucine motif L_799_L_800_ immediately upstream from the Y_802_W_803_ diaromatic motif, has been found to interact with prohibitin 1/2 (Phb) [249]. These proteins are produced almost ubiquitously; they are localized in numerous cellular compartments including mitochondria, the plasma membrane PM and the nucleus, and are also associated with lipid rafts. Disruption of the gp41-CT/Phb association, like gp41-CT truncation mutants, impairs cell-to-cell transmission in ‘non-permissive’ cells [249] (Figure 3 and 4). It is however not known whether this association genuinely contributes to the cell-to-cell transmission capacity of diverse HIV Envs or whether they are passively bound to gp41 at the time of assembly into lipid rafts. The L_799_L_800_ motif is not conserved between subtypes and is in many cases replaced by Val, particularly in subtype C. Nonetheless, in this work, the gp41-CT/Phb association was disrupted only when both Leu residues were mutated: single mutants were unaffected [249]. These findings suggest that this interaction, or interactions of this L_799_L_800_ motif with other cellular factors, is important enough to be preserved despite sequence variation. Moreover, this motif is also involved in interacting with p155-RhoGEF to block RhoA activation [250,251] (see section ‘Activation of transcription’ below), but there is no indication to date that these two paths converge.

### Activation of transcription

#### NF-κB

It was recently shown that the gp41-CT of HIV-1 activates NF-κB via the canonical pathway by interacting directly with TFG-β-activated kinase 1 (TAK1) [79]. TAK1 activates the transcription factors NF-κB and AP-1. TAK1 is itself activated by cytokines and microbial pathogens, and phosphorylates IκB, routing it for proteasomal degradation, thereby allowing NF-κB to translocate into the nucleus [252]. The gp41-CT/TAK1 interaction maps to the N-terminus of LLP-2 (C_764_-L_775_) and involves Y_768_ in the second YxxL motif [79] (Figure 3 and 4)_._ The HIV-1 LTR contains 2 (or 3, depending on subtype) NF-κB-binding sites [253]. The authors show that HIV mutants lacking the NF-κB-activating domains were more heavily dependent on cellular activation for replication, and thereby elegantly demonstrated that the gp41-CT contributes to viral replication in sub-optimally activated T lymphocytes by inducing the canonical NF-κB pathway [79]. In the case of SIV, the gp41-CT also activates the NF-κB pathway, but by acting upstream from TAK1, on a factor that has not yet been identified [31]. One of the functions of SIV and HIV Nef is to increase cellular activation in a manner similar to that induced by TCR engagement [90,228,254-257] in a NF-κB-independent manner [224,226,258,259]. Consequently, it has been suggested that Nef and Env may act in concert to progressively and steadily activate gene expression resulting in a positive feedback loop [79]. Alternatively, the early viral gene product Nef and the late gene product Env gp41-CT may act at different times or stages in the viral replication cycle, and induce different activation pathways [79], thereby fulfilling the requirement for two independent stimuli to fully activate T cells [260,261].

#### Luman

Luman (LZIP or CREB3) is a transcription factor belonging to the CREB/ATF family; it has been linked to the endoplasmic reticulum (ER) stress response. Luman activates the transcription of genes involved in ER-associated degradation of unfolded proteins [262-264]. The ER-membrane-bound form of luman is inactive; proteolytic cleavage releases an active form which enters the nucleus [263]. This activated form of luman interferes with Tat-mediated HIV transcription [265]. gp41-CT interacts with the ER-bound inactive precursor of luman, decreases its stability and targets it for degradation, thereby indirectly promoting LTR-mediated transcription (Figure 3). This interaction maps to residues 751–768, the region lying between the KE and LLP-2 [265] (Figure 4), and partly overlaps the gp41-CT region that interacts with TAK1 (C_764_-L_775_) [79]. Whether these two mechanisms somehow overlap or converge has not been explored.

#### P115-RhoGEF

Activation of RhoA through p155-RhoGEF inhibits HIV replication by affecting gene expression [251]. The gp41-CT of HIV interacts with p155-RhoGEF and thereby relieves the RhoA-induced inhibition [250,251]. This interaction involves L_799_ in LLP-3. L_799_ is highly conserved, and is part of the dileucine motif interacting with Phb1/2 [249] which tolerates polymorphic changes (Figure 4). Mutations in gp41-CT leading to loss of interaction with p115-RhoGEF impair HIV-1 replication in the human Jurkat T cell line (‘non-permissive’ to gp41-CT truncations) [250,251]. p155-RhoGEF is a specific guanine nucleotide exchange factor (GEF) involved in actin cytoskeletal organization rearrangements [250] through activation of RhoA GTPase, which is in turn involved in actin stress fiber organization, cell cycle progress through G1 and cell proliferation. Nonetheless, the gp41-CT-mediated relief of RhoA-mediated viral inhibition seems to be independent of the stress fiber formation function of RhoA and to involve other, as yet unknown, RhoA-mediated mechanisms [251]. Furthermore, whether this viral inhibition also applies in ‘permissive’ T cells or whether it is restricted to ‘non-permissive’ is not known: both relevant studies used ‘non-permissive’ cells. Comparisons between ‘permissive’ and ‘non-permissive’ cells may provide some clues as to the mechanism of action of this cellular factor in the HIV replication cycle.

### Immune evasion: countering intrinsic restriction/countering antiviral restriction factors

#### Tetherin

Tetherin (BST-2 or CD317) is an interferon-inducible transmembrane protein that restricts the release of nascent viral particles from infected cells by tethering them to the PM [266,267] reviewed in [268,269]. HIV-1 counteracts tetherin activity through Vpu (HIV-1) [266,267] which enhances ESCRT-dependent sorting for lysosomal degradation [270,271], whereas SIV Nef [272], and the HIV-2 Env ectodomain [273] counteract tetherin by intracellular sequestration through internalization motifs. One recent study reported that a Nef-deleted SIV strain acquired compensatory changes within the gp41-CT that restored resistance to rhesus tetherin through direct gp41-CT/tetherin interaction that sequestered tetherin [272]. The alterations of the SIV gp41-CT involved a tyrosine-based internalization motif similar to the HIV-2 gp41-CT [272], underlining the importance of countering tetherin and of Env trafficking for lentiviral replication. No such evolution has been reported for HIV-1.

#### Open questions

Numerous cellular proteins have been described to interact with the gp41-CT. Some of these proteins and interactions have been studied in detail, particularly those involved in Env internalization and trafficking through the TGN. However, in many cases, the role or the mechanisms of these interactions have not been documented. In some cases, the results and interpretations are controversial, and raise questions about whether the proteins are really involved in the viral replication cycle as active partners, or whether they are passively incorporated into the assembly platform, where they make only a limited if any contribution to viral replication. Different studies have been performed using different cell types and cell lines, each with their specific characteristics. Thus, there are issues about whether they reflect phenotypes of cell types relevant to the HIV replication cycle *in vivo*. Similarly, do these experimental models reflect the context of cell-to-cell transmission where the vicinity of cells and cell contacts are determinant parameters of viral infection and transmission? Some motifs involved in Env internalization and trafficking, as well as those involved in interaction with lipid rafts and in enhanced viral gene expression, are highly conserved across subtypes; others seems to display non-negligible sequence variation. It would be useful to assess whether such apparent tolerance identifies interactions that are less vital for viral replication, or whether the virus has acquired other compensatory polymorphisms to override decreased efficiency of that particular interaction.

## Conclusions

Initially considered to be inessential for the HIV replication cycle, the gp41-CT has now been demonstrated to be a pleiotropic domain: it is required for viral infectivity by ensuring Env incorporation into the virion on one hand, and contributing to enhanced viral replication in target cells through many still incompletely understood interactions on the other. Work on the gp41-CT has revealed that lentiviral assembly relies on finely coordinated interactions between diverse viral components subverting cell trafficking and activation factors. The virus thereby optimizes the infectious capacity of the neo-synthesized particle, prevents immune responses and preserves the virus-producing host cell until viral release. The uncontrolled release of immature virions would not only hinder viral propagation and be deleterious to the long-term maintenance of viral infection, but would also provoke a series of bystander effects, including Env-induced apoptosis, Env-mediated disruptive effects, and sensitivity to humoral immune responses.

The Env expressed on the surface of infected cells and of virions is one of the most powerful escape mechanisms developed by HIV. Its extreme variability, dense glycosylation and its very efficient cell-to-cell spreading capacity ensure extremely effective virus shielding. This shielding is further strengthened by the only weak expression of Env at the surface of infected cells and of virions, resulting from its late expression during the viral replication cycle, intense cycling and down-regulation through the TGN. Interference with cellular trafficking factors further contributes to viral masking. These latter functions are all fulfilled by the gp41-CT. Indeed, despite some sequence divergence between subtypes, structural motifs and some sequences motifs in the gp41-CT are strongly conserved. In particular, elements involved in Env trafficking from the plasma membrane through the endocytic and TGN compartments are conserved, underscoring their importance for viral replication and propagation. Other motifs involved in protein-protein interactions are not conserved providing evidence of subtype-related specificities. Such differences between subtypes have been poorly studied to date. The fine balance between sequence variability and highly controlled protein trafficking and dynamics allows the virus to optimize viral infectivity and viral propagation. The viral determinants that are poorly tolerant to polymorphic variation may well be interesting subjects for investigations to elucidate the subtle control of viral assembly, budding and release. Such work may lead to the identification of interactions that are possible targets for new antiviral strategies.

## Abbreviations

Ab: Antibody; AP: Adaptor protein; CA: Capsid; CaM: Calmodulin; CT: Cytoplasmic tail; Env: Envelope; ER: Endoplasmic reticulum; gp: Glycoprotein; HA: Hemagglutinin; HCV: Hepatitis C virus; HIV: Human Immunodeficiency Virus; IS: Inhibitory sequence; IκB: Inhibitor κB; KE: Kennedy epitope; LLP: Lentiviral lytic peptide; MA: Matrix; MPER: Membrane proximal external region; MSD: Membrane-spanning domain; MVB: Multi vesicular bodies; NF-κB: Nuclear Factor -κB; PM: Plasma membrane; PNG: Potential N-glycosylation site; PR: Protease; RER: Rough endoplasmic reticulum; SIV: Simian Immunodeficiency virus; SP: Spacer protein; TAK-1: TGF-β-interacting factor 1; TCR: T cell receptor; TGN: trans Golgi network; TIP47: Tail-interacting Factor 47 kDa; VSV-G: Vesicular stomatitis virus-glycoprotein.

## Competing interests

The authors declare no competing interests.

## Authors’ contributions

ESS, MM and DPB drafted the manuscript. All authors read and approved the final manuscript.
